# Effect of Surface Contamination on Near-Infrared Spectra of Biodegradable Plastics

**DOI:** 10.3390/polym16162343

**Published:** 2024-08-19

**Authors:** Namrata Mhaddolkar, Gerald Koinig, Daniel Vollprecht, Thomas Fruergaard Astrup, Alexia Tischberger-Aldrian

**Affiliations:** 1 Chair of Waste Processing Technology and Waste Management (AVAW), Montanuniversitaet Leoben, Franz-Josef-Strasse 18, 8700 Leoben, Austria; n.mhaddolkar@stud.unileoben.ac.at (N.M.);; 2 DTU SUSTAIN, Department of Environmental and Resource Engineering, Technical University of Denmark (DTU), Bygningstorvet, Bygning 115, 2800 Kongens Lyngby, Denmark; thap@ramboll.com; 3 Chair of Resource and Chemical Engineering, University of Augsburg, Am Technologiezentrum 8, 86159 Augsburg, Germany; daniel.vollprecht@uni-a.de; 4 Ramboll, Hannemanns Allé 53, 2300 Copenhagen S, Denmark

**Keywords:** biodegradable plastics waste management, near-infrared sorting, surface contamination, thermoplastic starch, effect on NIR spectrum, polylactic acid

## Abstract

Proper waste sorting is crucial for biodegradable plastics (BDPs) recycling, whose global production is increasing dynamically. BDPs can be sorted using near-infrared (NIR) sorting, but little research is available about the effect of surface contamination on their NIR spectrum, which affects their sortability. As BDPs are often heavily contaminated with food waste, understanding the effect of surface contamination is necessary. This paper reports on a study on the influence of artificially induced surface contamination using food waste and contamination from packaging waste, biowaste, and residual waste on the BDP spectra. In artificially contaminated samples, the absorption bands (ADs) changed due to the presence of moisture (1352–1424 nm) and fatty acids (1223 nm). In real-world contaminated samples, biowaste samples were most affected by contamination followed by residual waste, both having altered ADs at 1352–1424 nm (moisture). The packaging waste-contaminated sample spectra closely followed those of clean and washed samples, with a change in the intensity of ADs. Accordingly, two approaches could be followed in sorting: (i) affected wavelength ranges could be omitted, or (ii) contaminated samples could be used for optimizing the NIR database. Thus, surface contamination affected the spectra, and knowing the wavelength ranges containing this effect could be used to optimize the NIR database and improve BDP sorting.

## 1. Introduction

Biodegradable plastics, which are often advertised as an environmentally friendly alternative to conventional plastics, are gaining momentum in the global plastic market [1,2,3,4]. They form a 52% share of the total bioplastics produced in 2023 (amounting to 1.136 million tonnes) and are forecasted to grow to 4.605 million tonnes by 2028 [5]. This growth in production capacities necessitates that biodegradable plastics are recycled in order to accrue their environmental benefits [6].

For a product to be successfully recycled, it should be properly collected and correctly sorted [7,8]. In the modern waste management system, there are numerous methods available for sorting plastic wastes, for instance, density separation and sensor-based sorting [7]. Amongst these methods, near-infrared (NIR) sensor-based sorting is a widely applied method for sorting plastics in the waste industry [9,10,11,12]. It is a non-destructive method for sorting materials based on the irradiated NIR light, which is reflected from the waste material, and the reflected light contains spectral information to identify the material [10,13]. However, this material classification is dependent on the penetration depth of the NIR radiation [14,15]. Plastic waste has considerable surface contamination owing to its varied packaging applications (food and non-food) [16]. Thus, to use NIR technology for sorting plastic waste, it is necessary to study the effects of surface contamination on the spectra and, ultimately, their sorting [13,17,18].

Common applications of biodegradable plastics are food packaging, takeaway single-use food and beverage containers, supermarket carrier bags, and biowaste collection aids [19,20,21,22,23,24]. In other words, biodegradable plastics discarded by consumers are mostly food-related packaging and they are likely contaminated by food waste [25]. Moreover, of the few available studies discussing the NIR sortability of biodegradable plastics, almost all studies discuss only clean biodegradable plastics [26,27,28,29,30]. Nevertheless, knowing the effect of contamination on the spectra of these plastics is necessary for their effective sorting, which is not covered in the existing literature.

The present paper aims to fill this knowledge gap by studying the effect of surface contamination on the spectra of biodegradable plastics, with a focus on the state-of-the-art waste management infrastructure. The aim was to identify if the contaminants affected a specific wavelength range of the NIR spectra, and this information can be used to improve the sorting of biodegradable plastics. This assessment was conducted based on the following: (i) artificially induced contamination under laboratory conditions to account for the effect of common food contamination on the spectra and (ii) real-world contamination, to account for the effect of contamination from three waste streams on the spectra and the similarities/differences between them. In both cases, wavelength ranges undergoing drastic changes in the spectrum were identified. These findings will contribute to better implementation of the NIR sorting of biodegradable plastic waste and, ultimately, its recycling.

## 2. Materials and Methods

### 2.1. Experiment 1: Artificially Induced Contamination under Laboratory Conditions

#### 2.1.1. Samples, Used Contaminants, and Sample Preparation

**Figure 1:**
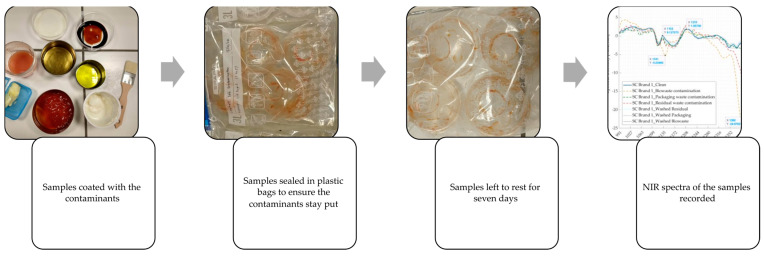
Steps followed for conducting experiment 1. Artificially induced contamination under laboratory conditions.

Two types of biodegradable plastic products (privately sourced by either procuring directly from the manufacturers [PLA lid] or collected from the items bought in day-to-day life [wood-fibre net]) were used for the present experiment (Table 1). Each sample of these biodegradable plastic products was coated with equal quantities (10 g) of eight contaminants, namely, (i) *beer*, (ii) *butter*, (iii) *curd*, (iv) *juice*, (v) *ketchup*, (vi) *olive oil*, (vii) *soya sauce*, and (viii) *water*. Each of these samples was sealed in a plastic airtight bag and left for seven days to let the contamination settle before recording their NIR spectrum data (Figure 1).

#### 2.1.2. Equipment Used as Well as Recording and Processing of Spectra

A lab-scale near-infrared sensor-based sorting equipment of Binder + Co AG was used to record the NIR spectrum (a hyperspectral image cube) of each of the clean and contaminated products. This equipment employed a EVK Helios NIR G2-320, Hyperspectral Imaging System, with a 930–1700 nm wavelength range. The hyperspectral imaging camera came with a spatial resolution of 312 effective pixels and a frame acquisition speed of 476 Hz at 220 spectral points. The following pre-processing techniques were employed in the raw spectrum for the analysis: spatial correction, intensity calibration, bad pixel replacement, noise suppression, first derivative, smoothing, and normalisation. The NIR light emitter was made up of an 800 W halogen lamp (HeLn Dr. Fischer 15026Z with a reflector). EVK Helios Optimizer Sqalar software (version 4.6.2019.2) was used for obtaining the hyperspectral image cube of the raw spectral data with 220 points, spaced equidistantly over the respective wavelength range, which was used for conducting principal component analysis (PCA). All necessary computations were conducted by using MATLAB by The MathWorks (Natick, MA, USA), Version 9.10.0.1710957 (R2021a) Update 4, on a Windows 10 computer equipped with an Intel^®^ UHD Graphics 630 and an Intel ^®^ Core ™ i5-9400H CPU clocked at 2.50 GHz.

**Figure 2:**
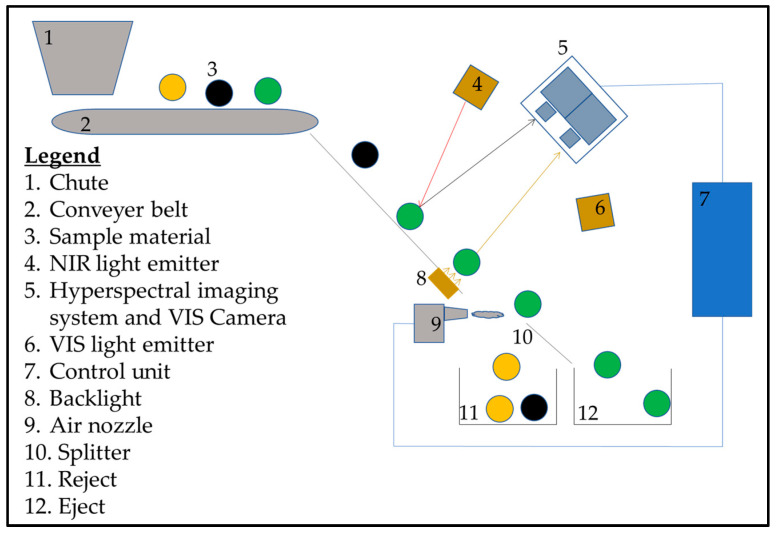
Chute-type near-infrared sorter set-up in the lab in Montanuniversitaet Leoben. NIR—Near infrared; VIS—Visible.

Figure 2 below shows the schematic for the chute-type NIR setup used for this experiment [31]. The emitted NIR light by the NIR light emitter (4) interacts with the sample material (3). Depending on the absorbed or reflected radiation, an NIR spectrum, colloquially called a fingerprint, is produced and reflected by the sample material (3). This radiation, which is recorded by the hyperspectral camera (5), is used to form a hyperspectral data cube. This cube is analysed by the control unit (7), which decides whether the material should be sorted out (ejected), depending on the settings and the NIR database. On receiving a ‘to-be-sorted’ signal from the control unit, the position of the material is obtained from the detected ‘absence of light’ by the VIS camera (5), and the air nozzle (9) is activated, thus ejecting the sample (12). For the present experiment, the operation was limited to generating the hyperspectral data cube of the sample. The PCA was conducted to compare the mean spectra of the contaminated and clean samples in the case of experiment 1 and the mean spectra of contaminated, washed, and clean spectra in the case of experiment 2.

### 2.2. Experiment 2: Real-World Contaminated Samples from the Three Waste Streams

#### 2.2.1. Sampling of Items and Sample Preparation

The samples for this experiment were obtained from the manual sampling analysis conducted in an Austrian district, where biodegradable plastics were manually sorted from packaging waste, biowaste, and residual waste streams using the visual identification method (using compostability labels and resin identification number 7 (Other) with PLA specified [32]). The samples were collected as part of the manual sorting trials conducted to identify the kind of biodegradable plastics present in the three waste streams, namely, packaging waste, biowaste, and residual waste streams [33]. The samples from the packaging waste were collected from the rejected fraction of a material recovery facility, while those from biowaste were collected from the input fraction of an industrial compost facility. Lastly, the samples from residual waste were collected from the input fraction to a splitting plant. The detailed sampling methodology is described in [33]. From the gathered samples, three biodegradable supermarket carrier bags (SC) from 3 brands were selected, which were present in all three waste streams—supermarket carrier bags from brand 1 (SC Brand 1), SC Brand 2, and SC Brand 3. Using a Fourier transform infrared (FTIR) spectrometer (Cary 630 from Agilent Technologies with a single reflection diamond), the clean samples of the three supermarket carrier bags were identified as a blend of thermoplastic starch, polybutylene adipate terephthalate, and polycaprolactone (brand name: Mater-Bi [34]).

**Figure 3:**
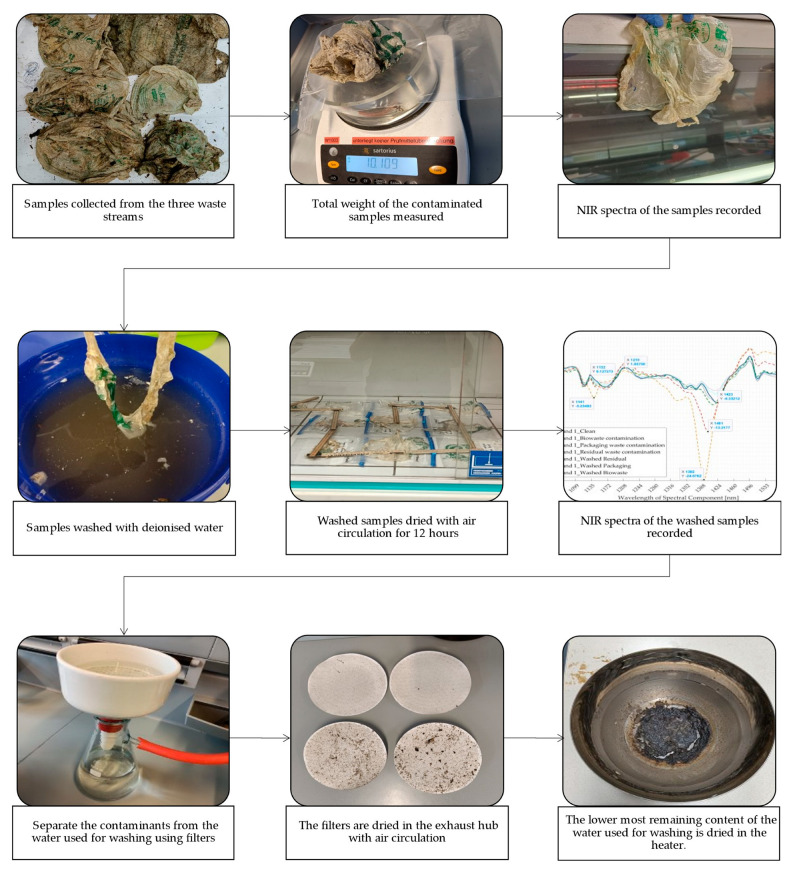
Steps for conducting experiment 2: Contaminated samples from packaging waste, biowaste, and residual waste streams. NIR—Near infrared.

To determine the degree of contamination of the samples from the three different waste streams, the samples were washed three times using deionised water (0.75 L). While washing, the samples were moved around in water, soaked for some time, and moved again; this combination of soaking and moving was to facilitate the removal of maximum contaminants from the surface. Next, these samples were dried for 12 h using air circulation in an exhaust hood. The contaminants from the water used for washing were filtered out using a membrane filter with a pore diameter of 12 µm. These used filters were dried using air circulation in the exhaust hood, and the weight of the contaminants was calculated by subtracting the dried filter’s weight from the tare weight of the filters. Additionally, after filtering about 90% of the dirty water, the remaining ~10% residue was dried in a lab oven (Memmert UF 110) at 105 °C. The weight of these dried contaminants plus that of contaminants from the dried filter gave the value of total contaminants from the respective waste stream. Figure 3 describes the steps taken for experiment 2.

#### 2.2.2. Equipment Used as Well as Recording and Processing of Spectra

The same equipment described in Section 2.1.2 was used for this experiment.

## 3. Results and Discussion

The effect of surface contamination on the product spectra is presented in this section for the two different experimental approaches.

### 3.1. Experiment 1: Artificially Induced Contamination Using Food-Derived Contaminants

The spectrum of the clean biodegradable plastics item was compared to that of the artificially induced contaminated samples (using eight food waste contaminants) of PLA lid and wood fibre net samples (Table 1). The effect of contamination was observed as a change in the intensity and shape of the spectral absorption bands (first derivative) at certain wavelength ranges.

**Figure 4:**
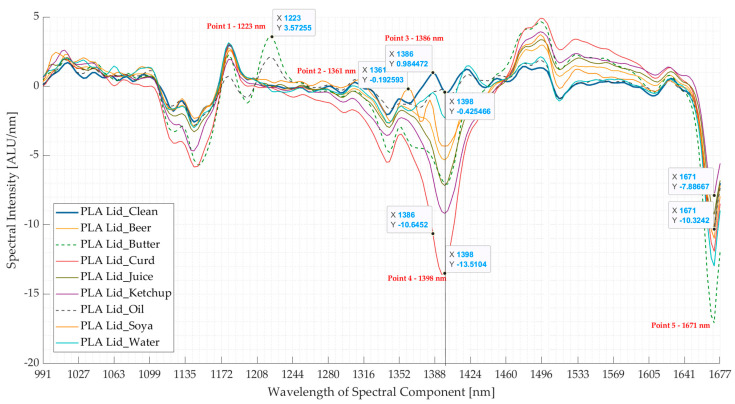
Comparing the mean spectra (first derivative) of clean polylactic acid (PLA) lid with 8 surface contaminated PLA lids’ spectra. The 8 surface contaminants used were beer, butter, curd, juice, ketchup, oil, soya, and water. Five points were selected (point 1 at 1220 nm, 2 at 1361, 3 at 1386 nm, 4 at 1398 nm, and 5 at 1671 nm) to highlight the change in spectra due to contamination. ALU—Arbitrary light units; nm—nanometre.

In most cases, the presence of surface contaminants resulted in an alteration of the intensity of the absorption bands (Figure 4). Five wavelength points were selected to highlight major changes because they demonstrated a clear distinction between the spectra of most of the contaminated and clean samples.

The *beer-*contaminated sample had its NIR spectrum (first derivative) closely following that of the clean sample, except for the changed intensity of the absorption band at point 4 (1398 nm) and point 5 (1671 nm), and omission of the absorption band at point 3 (1386 nm). Similar behaviour was observed for the spectra of *curd-*, *juice-*, and *ketchup-*contaminated samples, where the *curd*-contaminated sample’s spectrum had the highest change in intensity. The NIR spectrum of the *water-*contaminated sample also presented the same behaviour, except for the omission of absorption bands at point 3. On the other hand, the *soya*-contaminated sample had a shift in the absorption band peak from 1351 nm to 1361 nm (point 2) in its spectrum, while for the rest the changes were the same as in the *beer-*contaminated sample’s spectrum. In the case of the spectrum of the *butter-*contaminated sample, there was an additional absorption band at point 1 (1223 nm), and here again, the rest of the changes were the same as demonstrated by the spectrum of the *beer*-contaminated sample. This additional peak at point 1 was also present in the spectrum of the *oil*-contaminated sample; however, for the rest of the points, the change in the spectrum was not as pronounced as in the spectra of the other contaminated samples. The presence of this additional absorption band at point 1 can be attributed to the presence of fatty acids (C-H stretching) [35,36,37,38]. The effect of moisture was observed with an increased intensity of the absorption bands at point 4 (1398 nm) for seven contaminated samples [39], except for the *oil-*contaminated sample. In general, for all the eight contaminant samples, there was mostly a change in the intensities of the spectral absorption bands at wavelengths of 1099–1172 nm and 1460–1677 nm, whereas there was a change in spectral shape at 1352–1424 nm (and 1188–1244 nm for *butter* and *oil*). Thus, Figure 4 shows that surface contamination affects the spectrum of the material at certain wavelength ranges.

**Figure 5:**
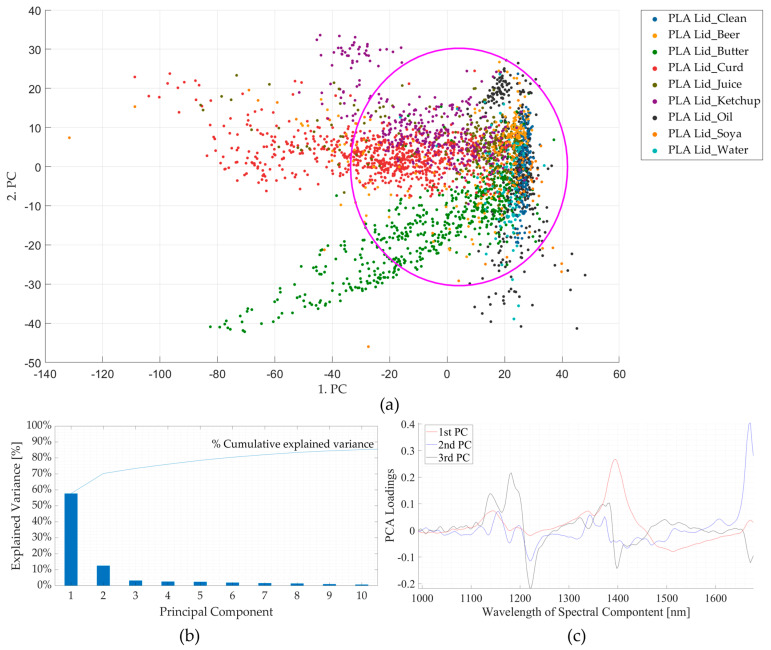
Results of principal component analyses for clean and contaminated PLA lid samples. (**a**) Comparison between the first and second principal components. (**b**) Pareto plot showing the contribution of individual principal components to the total variance and cumulative variance. The first three components are seen to contribute to ~80% variance. (**c**) Loading plots for the first three principal components (PC). nm—nanometre; PLA—polylactic acid.

The effect of contamination is also demonstrated in the PCA of NIR spectra for clean PLA versus those of surface-contaminated samples. From Figure 5b, it is seen that the first three components contribute to ~80% of the total variance. The absorption bands contributing to maximum variance in the three principal components (PCs) are shown in Figure 5c, which coincide with the five points selected in Figure 4. From the score plots (PC1 *v*/*s* PC2) shown in Figure 5a, it is seen that part of the points are clustered around the *clean* PLA sample, which could be due to the uncontaminated surface of those samples. Meanwhile, some of the points of the contaminated samples appear to spread in an outward direction from the cluster, which could be because of the contaminated surface as they do not cluster near the points of the *clean* sample. This can be seen in the case of the *butter-*contaminated sample, where some points are seen to be clustered around the clean sample region (uncontaminated surface), while most of the points spread outwards (contaminated surface) from this cluster marked by a magenta circle. Thus, the presence of surface contamination influences the spectra, which is also reflected in the PCA score plots.

**Figure 6:**
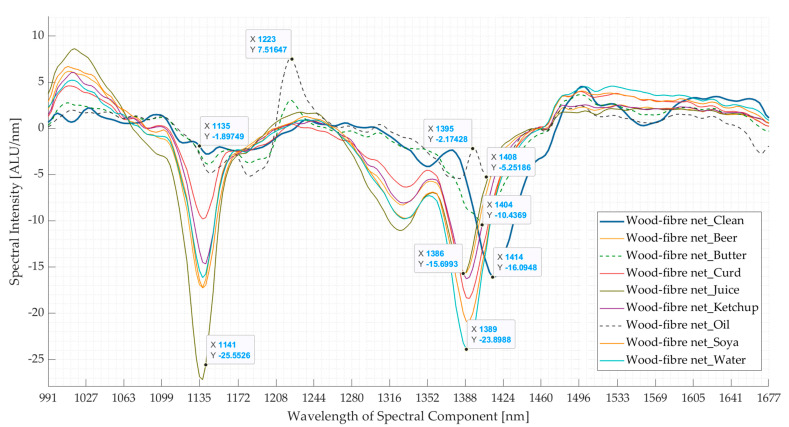
Comparing the mean spectra (first derivative) of the clean wood-fibre net with 8 surface-contaminated wood-fibre nets’ spectra. The 8 surface contaminants used were beer, butter, curd, juice, ketchup, oil, soya, and water. ALU—Arbitrary light units; nm—nanometre.

**Figure 7:**
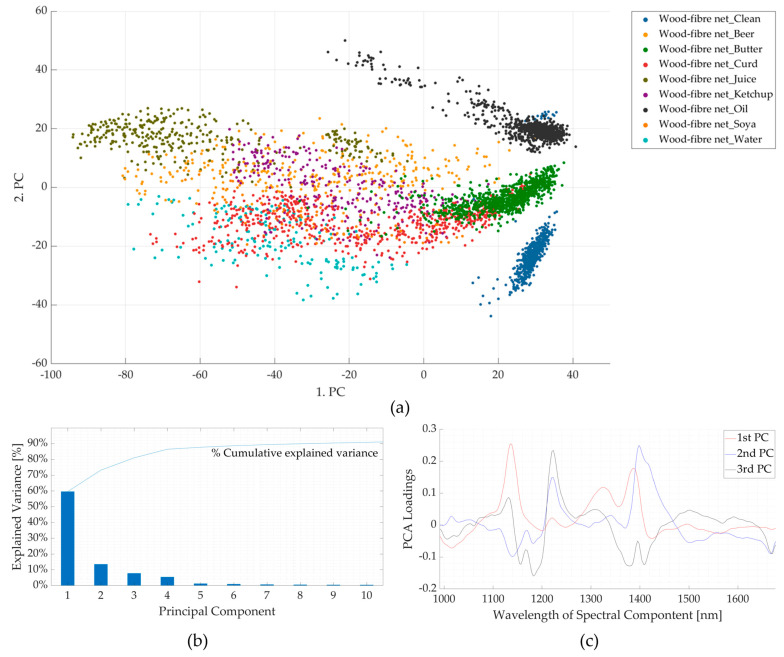
Results of principal component analyses for clean and contaminated wood-fibre net samples. (**a**) Comparison between the first and second principal components. (**b**) Pareto plot showing the contribution of individual principal components to the total variance and cumulative variance. The first three components are seen to contribute to ~80% variance. (**c**) Loading plots for the first three principal components (PC). nm—nanometre.

In the case of the wood-fibre net, the effect of contaminants was more pronounced, possibly because the material soaked the contaminants in contrast to the PLA lid sample. The mean spectra of the clean and contaminated samples are shown in Figure 6. The absorption band at 1414 nm of the clean sample spectra (first derivative) was shifted to 1386–1389 nm for *beer-*, *curd-*, *juice-*, *ketchup-*, *soya-*, and *water*-contaminated samples, to 1404 nm for *butter*-contaminated samples, and to 1408 nm for *oil*-contaminated samples. Except for the *oil*-contaminated samples, this shift in wavelength of the seven contaminated samples could be attributed to the presence of moisture [39]. Another difference is that the absorption bands between 1135 and 1141 nm, which is attributed to the lignin content [40,41,42], were more pronounced in the presence of contaminants, except for *butter-* and *oil*-contaminated samples, which followed the *clean* sample. These two changes contribute to maximum variance in PC 1. Lastly, as in PLA lid sample results, the presence of fatty acids resulted in the additional absorption band at 1223 nm [35,36,37,38]. The first two changes contribute to ~60% of the total variance (PC1), whereas the combination of the first three PCs contributes to ~80% of the total variance, as seen in Figure 7b,c. Thus, the effect of contamination is more pronounced on the mean spectra of the wood-fibre nets than in the PLA lid. This is also demonstrated by the PCA score plots (PC1 v/s PC2) in Figure 7a, where the points of the *clean-*, *butter-*, and *oil*-contaminated samples have almost separate clusters, whereas it is difficult to differentiate the points of the remaining six contaminated samples, although on close observation a pattern can be seen for each contaminant. This shows that when the sample is soaked with the contaminants, the effect of these contaminants on the spectra is more pronounced.

This effect of surface contamination on the spectra could potentially affect the sorting of biodegradable plastics if the NIR database uses a training set consisting of only clean samples. Moreover, the different contamination has distinct PCA score plots (and spectra), although the changes could be due to only changes in the intensity of the first derivative spectra. Therefore, by knowing the effect of the contaminants on the spectra, the NIR database could be optimized to effectively sort the biodegradable plastics [33]. The knowledge of the wavelengths which are principally affected by the contaminants could assist in selecting the remaining ranges which are independent of the effect of contaminants. For example, the wavelength range of 1208–1236 nm is seen to be affected by the presence of fatty acids, whereas 1352–1424 nm is seen to be affected by moisture content.

From Figure 4, Figure 5c, Figure 6 and Figure 7c, the wavelength ranges heavily affected by the surface contamination can be obtained. To improve the NIR database, two different approaches can be taken: (i) exclude the wavelength ranges which are explicitly affected by the contamination, or (ii) update the NIR database using the training sets including contaminated samples. Although the first approach seems simpler than the second, it can lead to omitting important absorption band information, as there might not be enough characteristic spectral information left to make a correct identification decision, ultimately leading to incorrect sorting. On the other hand, even if the second approach is time-consuming, it would lead to better sorting results. Thus, updating the NIR database with the training sets of contaminated samples (for instance, by clustering the contaminated and clean samples together in Figure 5a and Figure 7a) will improve sorting and avoid false positives/negatives. In addition to the second approach, instead of completely excluding the affected wavelength ranges, lower weightages could be applied to these ranges while considering the sorting algorithm. This will ensure that the important absorption bands occurring at the wavelength ranges are considered but with a lower weightage. In other words, the NIR machine will give more weightage to the other wavelength ranges while making the sorting decision instead of the ranges heavily affected by surface contamination.

### 3.2. Experiment 2: Contaminated Samples from the Three Waste Streams

Firstly, from the washing experiment, the level of contamination of samples from three waste streams was calculated (Table 2). Based on weight, samples from biowaste had a greater weight of contaminants than the samples from packaging and residual waste, which could be attributed to the high moisture content in the contamination. Additionally, on visual observation, biowaste samples were more covered by the contamination, followed by residual waste samples, whereas the packaging waste sample had the least level of contamination. However, based on the %weight share of contaminants from the total sample weight, residual waste samples were found to have the maximum value (21%), followed by biowaste samples (20%), while the samples from packaging waste had the least value (18%); thus, the contamination level did not differ drastically between the samples from the three waste streams. The average %weight of contaminants was calculated to be 20%.

**Figure 8:**
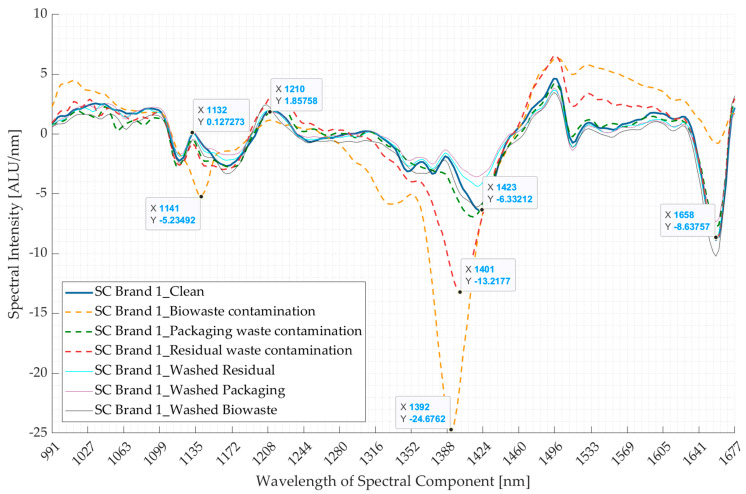
Comparing the mean spectra (first derivative) of clean supermarket carrier bag brand 1 (SC Brand 1) with that of the contaminated samples from packaging waste, biowaste, and residual waste and the spectra of these samples after washing. ALU—Arbitrary light units; nm—nanometre.

**Figure 9:**
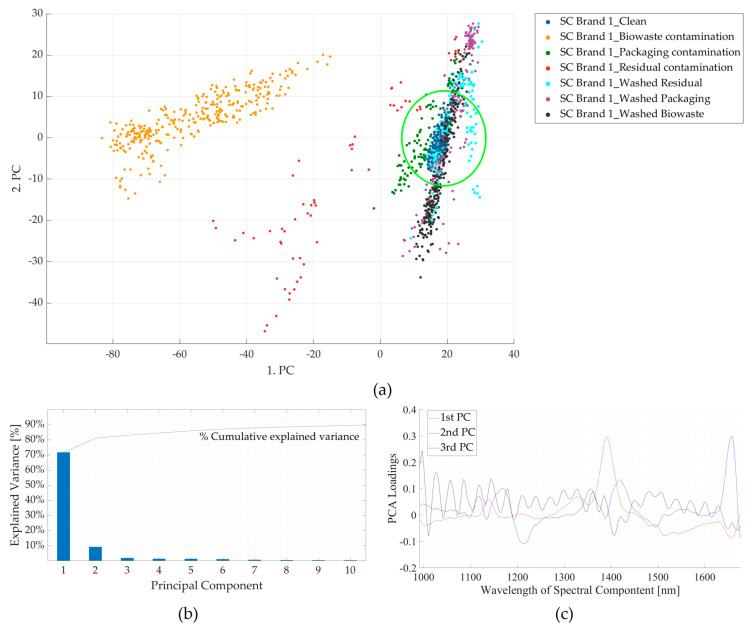
Results of principal component analyses of clean and contaminated samples of supermarket carrier bag brand 1 (SC Brand 1). (**a**) Comparison between the first and second principal components. (**b**) Pareto plot showing the contribution of individual principal components to the total variance and cumulative variance. The first three components are seen to contribute to ~80% variance. (**c**) Loading plots for the first three principal components (PCs). nm—nanometre.

This section discusses the results for supermarket carrier bag brand 1 (SC Brand 1), while the results for SC Brand 2 and SC Brand 3 are included in Appendix A. Figure 8 shows the comparison of spectra of SC Brand 1 for clean, contaminated (from packaging waste, biowaste, and residual waste), and washed samples. The spectra of SC Brand 1 with biowaste and residual waste contaminants are seen to have significant changes, whereas the spectra of packaging waste and washed samples closely follow the spectra of clean samples with slight changes in intensities of absorption bands (first derivative) (Figure 9a).

The effect of contamination was strongly observed in the samples from biowaste, followed by that from residual waste, and least in the packaging waste. The change in spectra between 1352 and 1424 nm could be attributed to the moisture content in the three contaminated samples [39], which was more visible in biowaste than residual waste and least in the packaging waste-contaminated samples. This is also the first principal component (Figure 9c). Additionally, biowaste samples had a distinct absorption band at 1141 nm, which could be due to the presence of soil [43,44,45]. Washing the contaminated samples significantly removed the effect of contamination on the spectra, and their spectra only differed from that of the clean sample in terms of changed intensities of absorption bands, for instance, at 1423 nm and 1658 nm.

Figure 9 shows the results for the PCA of the NIR spectra for clean SC Brand 1 versus that of contaminated and washed samples. From Figure 9b, it is seen that the first three principal components contribute to ~80% of the total variance, and the absorption bands contributing to maximum variations in the three principal components are shown in Figure 9c. From the score plots (PC1 *v*/*s* PC2) shown in Figure 9a, it is seen that the points of clean, washed, and packaging waste-contaminated samples are clustered together (marked by a green circle), and those for residual waste-contaminated samples appear to spread in an outward direction from this cluster. However, the score plots for biowaste samples appear to be completely away from this cluster. Figure 9c shows that the PC1 is mainly due to a high variance at the wavelength range of 1352–1424 nm, which coincides with the more pronounced difference in the spectra of biowaste-contaminated samples, compared to the other contaminated, washed, and clean samples, which is seen in the resultant Pareto plot (Figure 9b) and the PCA score plots (Figure 9a). The results could be different if the biowaste-contaminated sample is excluded from the PCA, and, here, perhaps the differentiation between the results of packaging waste-contaminated, washed, and clean samples will be more evident, as PC1 will not be influenced by the variance between the biowaste-contaminated sample and these samples.

The above results can be summarised as follows: (i) biowaste-contaminated samples had a significantly different spectrum than the other samples, (ii) the packaging waste-contaminated spectra closely followed those of clean and washed samples, with a slight change in intensity, and (iii) the effect of contamination on spectra can be considerably removed by washing the samples.

**Figure 10:**
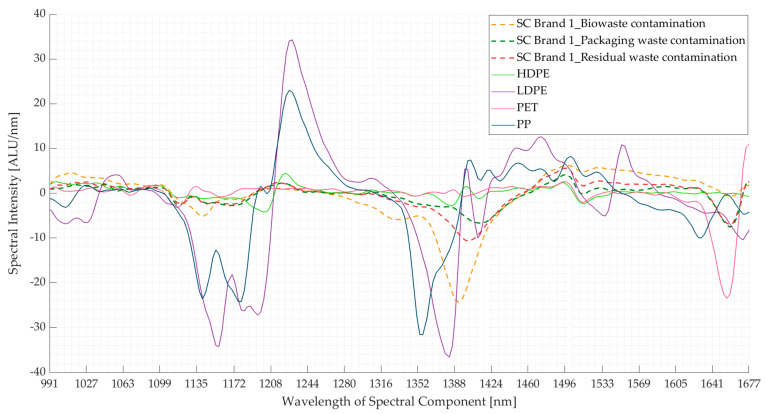
Comparing the mean spectra (first derivative) of three contaminated samples of supermarket carrier bag brand 1 (SC Brand 1) with high-density polyethylene (HDPE), low-density polyethylene (LDPE), polyethylene terephthalate (PET), and polypropylene (PP) spectra. ALU—Arbitrary light units; nm—nanometre.

As in real-world conditions, biodegradable plastics need to be sorted out of a mixture containing other plastics. The spectra of the contaminated biodegradable plastic samples were compared with those of four conventional plastics, namely, high-density polyethylene (HDPE), low-density polyethylene (LDPE), polyethylene terephthalate (PET), and polypropylene (PP) (Figure 10). It was found that the LDPE and PP spectra appeared different from the contaminated samples in comparison to the spectra of HDPE and PET (Figure 10). The wavelength ranges where the spectra of contaminated samples overlapped with those of HDPE and PET spectra at 1047–1115 nm, 1244–1272 nm, and 1549–1569 nm.

**Figure 11:**
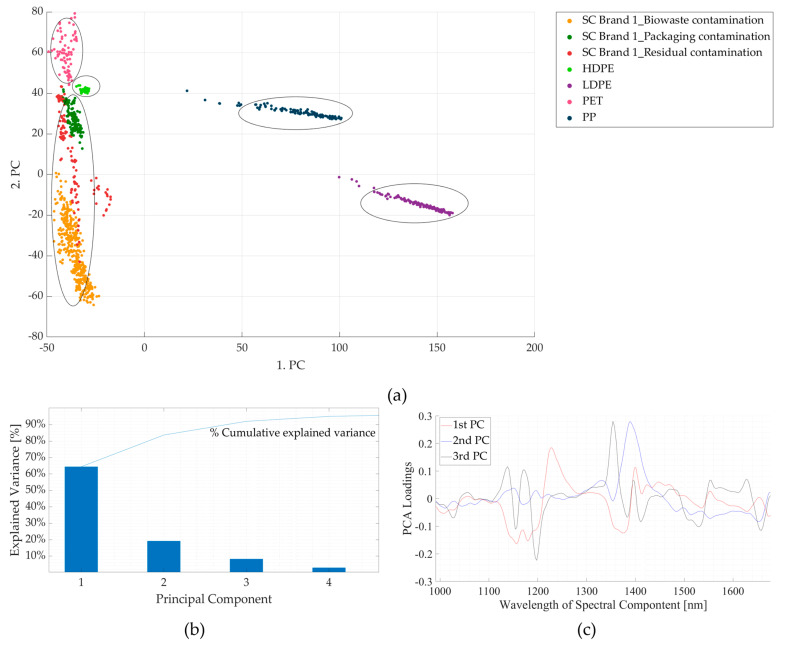
Results of principal component analyses of contaminated samples of supermarket carrier bag brand 1 (SC Brand 1) and four conventional plastics (HDPE, LDPE, PET, and PP). (**a**) Comparison between the first and second principal components. (**b**) Pareto plot showing the contribution of individual principal components to the total variance and cumulative variance. The first three components are seen to contribute to ~80% variance. (**c**) Loading plots for the first three principal components (PCs). HDPE—high-density polyethylene; LDPE—low-density polyethylene (LDPE); nm—nanometre; PET—polyethylene terephthalate; PP—polypropylene.

**Figure A1:**
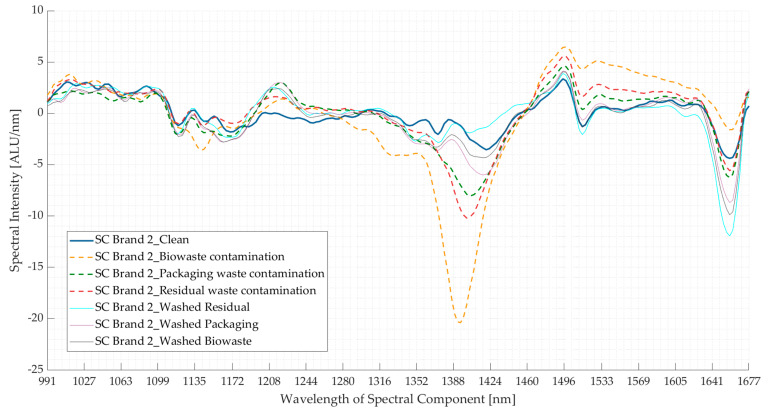
Comparing the mean spectra (first derivative) of clean supermarket carrier bag brand 2 (SC Brand 2) with those of the contaminated samples from packaging waste, biowaste, and residual waste and the spectra of these samples after washing. ALU—Arbitrary light units; nm—nanometre.

**Figure A2:**
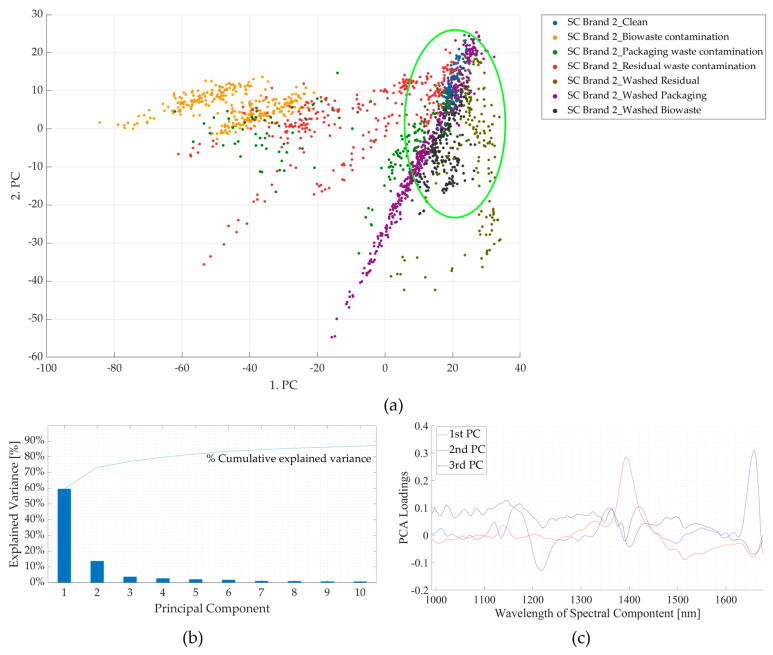
Results of principal component analyses of clean and contaminated samples of supermarket carrier bag brand 2 (SC Brand 2). (**a**) Comparison between the first and second principal components. (**b**) Pareto plot showing the contribution of individual principal components to the total variance and cumulative variance. The first three components are seen to contribute to ~80% variance. (**c**) Loading plots for the first three principal components (PCs); nm—nanometre.

**Figure A3:**
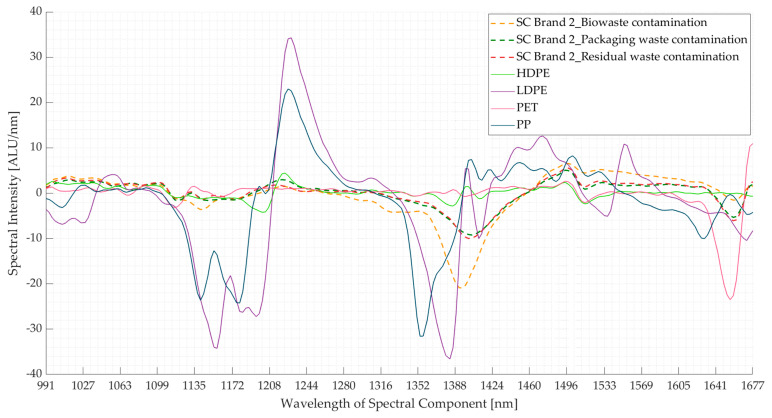
Comparing the mean spectra (first derivative) of three contaminated samples of supermarket carrier bag brand 2 (SC Brand 2) with high-density polyethylene (HDPE), low-density polyethylene (LDPE), polyethylene terephthalate (PET), and polypropylene (PP) spectra. ALU—Arbitrary light units; nm—nanometre.

**Figure A4:**
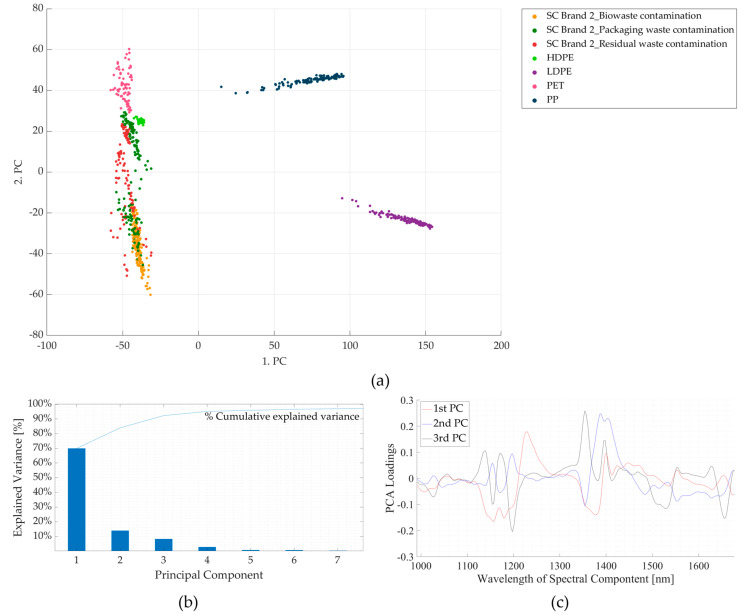
Results of principal component analyses of contaminated samples of supermarket carrier bag brand 2 (SC Brand 2) and four conventional plastics (HDPE, LDPE, PET, and PP). (**a**) Comparison between the first and second principal components. (**b**) Pareto plot showing the contribution of individual principal components to the total variance and cumulative variance. The first three components are seen to contribute to ~80% variance. (**c**) Loading plots for the first three principal components (PCs). HDPE—high-density polyethylene; LDPE—low-density polyethylene (LDPE); nm—nanometre; PET—polyethylene terephthalate; PP—polypropylene.

**Figure A5:**
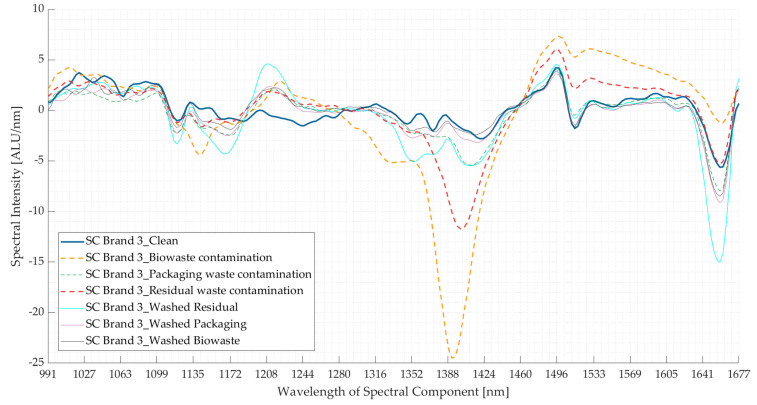
Comparing the mean spectra (first derivative) of clean supermarket carrier bag brand 3 (SC Brand 3) with that of the contaminated samples from packaging waste, biowaste, and residual waste and the spectra of these samples after washing. ALU—Arbitrary light units; nm—nanometre.

**Figure A6:**
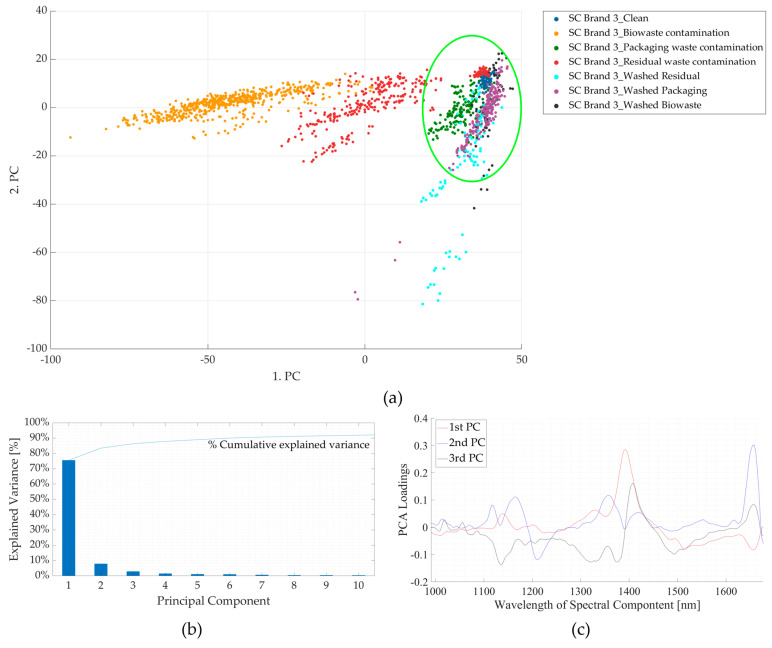
Results of principal component analyses of clean and contaminated samples of supermarket carrier bag brand 3 (SC Brand 3). (**a**) Comparison between the first and second principal components. (**b**) Pareto plot showing the contribution of individual principal components to the total variance and cumulative variance. The first three components are seen to contribute to ~80% variance. (**c**) Loading plots for the first three principal components (PCs). nm—nanometre.

**Figure A7:**
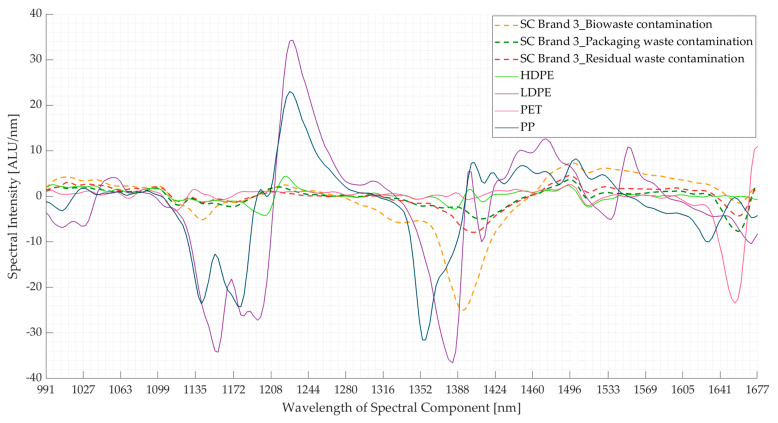
Comparing the mean spectra (first derivative) of three contaminated samples of supermarket carrier bag brand 3 (SC Brand3) with high-density polyethylene (HDPE), low-density polyethylene (LDPE), polyethylene terephthalate (PET), and polypropylene (PP) spectra. ALU—Arbitrary light units; nm—nanometre.

**Figure A8:**
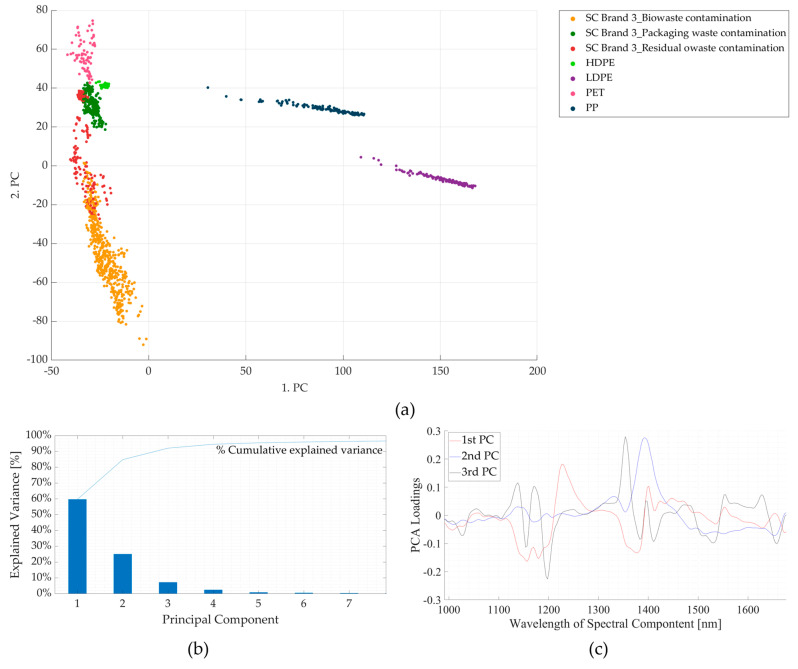
Results of principal component analyses of contaminated samples of supermarket carrier bag brand 3 (SC Brand 3) and four conventional plastics (HDPE, LDPE, PET, and PP). (**a**) Comparison between the first and second principal components. (**b**) Pareto plot showing the contribution of individual principal components to the total variance and cumulative variance. The first three components are seen to contribute to ~80% variance. (**c**) Loading plots for the first three principal components (PCs). HDPE—high-density polyethylene; LDPE—low-density polyethylene (LDPE); nm—nanometre; PET—polyethylene terephthalate; PP—polypropylene.

Figure 11 shows the results of the PCA of the NIR spectra for three contaminated samples of SC Brand 1 with the HDPE, LDPE, PET, and PP spectra. From Figure 11b, it is seen that the first three components contribute to ~80% of the total variance. From the score plots (PC1 *v*/*s* PC2) shown in Figure 11a, it is seen that the data points of LDPE and PP are positioned away from the three contaminated samples, whereas those of HDPE and PET are closer. The score plots of the contaminated samples follow the pattern seen in Figure 9a. The absorption bands contributing to maximum variance in the three principal components are shown in Figure 11c. Thus, surface contamination could pose a problem in sorting the contaminated thermoplastic starch blend from HDPE and PET, while it should not be a problem with LDPE and PP. The same behaviour is observed for the spectra of the other two contaminated samples, which are presented in Appendix A (Figure A1, Figure A2, Figure A3, Figure A4, Figure A5, Figure A6, Figure A7 and Figure A8).

The two approaches discussed in Section 3.1 are also applicable here, where the heavily affected wavelength ranges (Figure 8 and Figure 9c) could either be excluded completely or included in the NIR database with proper weightages applied, where necessary. In the first approach, excluding the contamination-influenced spectral regions (e.g., range 1368–1424 nm) could reduce the necessary spectral information for differentiating the contaminated samples from the HDPE and PET samples. However, excluding the spectral region between 1127 nm and 1151 nm could help prevent the overlap of the biowaste-contaminated sample with the PP sample spectra. Similarly, excluding spectral regions between 1208 and 1244 nm would prevent overlap with the *oil-*contaminated samples (Figure 4) and PET spectra (Figure 10). Nevertheless, the negative effects of excluding certain spectral ranges on the proper sorting are more as compared to the positive effects. The second approach was adopted while sorting biodegradable plastics from the three waste streams using an NIR sorter (explained in Section 2.2.1), where the NIR database was created using clean and contaminated samples [33]. However, this approach largely depends on the availability of the contaminated samples to optimize the NIR database.

Additionally, the results show that the spectra of samples from the waste streams are different from each other; therefore, the NIR databases created should be updated for the respective waste streams. In the case of biowaste samples, the position of the NIR sorter in the sequence of waste treatment processes also affects the spectra because sorting biodegradable plastics after they are mixed with soil will include an additional absorption band due to the presence of soil. Packaging waste had the least amount of contamination, and its effect on the spectra was minimal. Lastly, including an NIR sorter at a residual waste treatment facility does not make sense because the waste is ultimately sent for thermal recovery; nevertheless, this study tested the effect of contamination on the spectra to identify the similarities/differences between the spectra of the samples from three waste streams. From this, the wavelength ranges identified to be repeatedly affected by contamination were 1352–1424 nm and 1464–1671 nm in the case of the three contaminated samples, and 1123–1157 nm particularly in biowaste-contaminated samples. In all the cases, except for 1352–1424 nm, the characteristic peaks of the biodegradable plastic materials were present. Also, the contaminated samples’ spectra (and score plots) of HDPE and PET were found to be overlapping at certain wavelengths. This could lead to false classification of these conventional plastics if the NIR classifier relies heavily on these spectral ranges [13,46]. However, this could be taken care of by proper classification of the clusters (as shown in Figure 11a).

## 4. Conclusions

Biodegradable plastic applications could have heavy contamination of food waste, which might affect their NIR spectra and sortability. This paper focuses on studying the effects of surface contamination on the spectra of biodegradable plastics, which was conducted using (i) artificially induced contamination in samples using eight typical food waste and (ii) real-world contaminated samples collected from packaging waste, biowaste, and residual waste.

For all eight artificially induced contamination samples, the wavelength range of 1352–1424 nm demonstrated maximum alteration (mainly due to moisture presence), whereas ranges 1161–1258 nm and 1505–1589 nm demonstrated only change in intensity of absorption bands in the spectra (first derivative). However, *butter-* and *oil*-contaminated samples were an exception with a distinct new absorption band at 1223 nm due to fatty acids content. The effect of contaminants was more pronounced in wood-fibre net samples than PLA-lid because the former was soaked with the contaminants, while the latter had them on its surface. On the other hand, the spectra of contaminated samples from biowaste and residual waste strongly differed from the clean sample, while the spectra of packaging waste, washed, and clean samples were not drastically different from each other. The results showed that the wavelength ranges of 1352–1424 nm and 1464–1671 nm in the spectra of three contaminated samples, and 1123–1157 nm in the spectra of biowaste-contaminated samples, were affected by the presence of contamination. Additionally, the spectra of contaminated samples from three waste streams were compared with four conventional plastics to account for the real-world conditions. The spectra (first derivative) of LDPE and PP were different from the three contaminated samples, which is also demonstrated by the score plots comparing the first principal component to the second. On the other hand, the spectra of HDPE and PET overlapped at certain wavelength ranges (1047–1115 nm, 1244–1272 nm, and 1549–1569 nm), and their scores (points) appeared close to those of the contaminated samples but were still distinguishable.

Thus, the results demonstrated that surface contamination affected the spectra, and using the NIR database created only with the clean samples could result in false sorting. To negate the problems resulting from these observations, two approaches could be followed to consider the effects of surface contamination on the spectra of biodegradable plastics, and ultimately, their sorting. The first proposed approach was to exclude the wavelength ranges heavily affected by the contamination, especially with the drastic change in spectra, from the NIR database; however, this could result in the omission of important spectral information necessary for the material identification. The second proposed approach was to optimize the NIR database using the training sets containing the contaminated samples, which could lead to improved sorting of the biodegradable plastics. This approach could be combined with reduced weightage of the wavelength ranges with heavy contamination (e.g., at 1368–1424 nm). Thus, knowing the wavelength range where the effect of contamination is present will assist in developing a better NIR database and improve the sorting of biodegradable plastics, which will ultimately contribute to their recycling in a state-of-the-art waste management system.

## Figures and Tables

**Table 1:** Products and types of biodegradable plastics used for lab contamination experiments.

Sr. No.	Biodegradable Plastic Products		Type of Biodegradable Plastics
1	Single-use cup lid	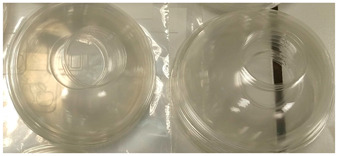	Polylactic acid
2	Vegetable packaging (net)	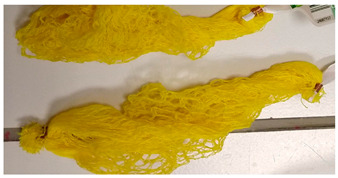	Wood fibre

**Table 2:** Contamination level of the samples from packaging waste, biowaste, and residual waste streams.

Source of Contaminated Samples	Contaminant Weight (g)	Total Sample Weight (g)	% Contamination
Packaging waste	4.770	26.094	18%
Biowaste	16.593	82.703	20%
Residual waste	4.243	20.271	21%

## Data Availability

Data are contained within the article.

## Appendix A

In the case of SC Brand 2 (Figure A1), the effect of contamination was strongly observed in the samples from biowaste, followed by those from residual waste, and unlike SC Brand 1, the effect of contamination observed in the packaging waste samples is more pronounced (in the 1352–1424 nm spectral region). The other results are in line with the SC Brand 1 results discussed in Section 3.2.

Figure A2 shows the results of the PCA of the NIR spectra for clean samples versus those of contaminated and washed samples of SC Brand 2 and SC Brand 3, respectively. From the score plots (PC1 *v*/*s* PC2) shown in Figure A2a, it is seen that the points of clean and washed samples are clustered together (marked by a green circle), and those for packaging waste-contaminated and residual waste-contaminated samples appear to spread in an outward direction from this cluster. However, the score plots for biowaste samples appear to be completely away from this cluster.

Figure A3 shows that LDPE and PP spectra appear different from the contaminated samples of SC Brand 2 in comparison to the spectra of HDPE and PET. Additionally, from the score plots (PC1 *v*/*s* PC2) in Figure A4a, it is seen that the data points of LDPE and PP are positioned away from the three contaminated samples, whereas those of HDPE and PET are closer. Thus, the results are in line with those of SC Brand 1.

The results for SC Brand 2 (Figure A5) were in line with those for SC Brand 1 discussed in Section 3.2; the effect of contamination was strongly observed in the samples from biowaste, followed by residual waste, and least in the packaging waste. From Figure A6a, it is seen that the points of clean, washed, and packaging waste-contaminated samples are clustered together (marked by a green circle), and those for only residual waste-contaminated samples appear to spread in an outward direction from this cluster, whereas the score plots for biowaste samples appear to be completely away from this cluster.

Figure A7 shows that the LDPE and PP spectra appear different from the contaminated samples of SC Brand 2 in comparison to the spectra of HDPE and PET. Additionally, from the score plots (PC1 *v*/*s* PC2) in Figure A8a, it is seen that the data points of LDPE and PP are positioned away from the three contaminated samples, whereas those of HDPE and PET are closer. Thus, the results are in line with those of SC Brand 1.
